# Change of Consumption Behaviours in the Pandemic of COVID-19: Examining Residents’ Consumption Expenditure and Driving Determinants

**DOI:** 10.3390/ijerph18179209

**Published:** 2021-08-31

**Authors:** Jimin Xiong, Zhanfeng Tang, Yufeng Zhu, Kefeng Xu, Yanhong Yin, Yang Xi

**Affiliations:** 1 Faculty of Maritime and Transportation, Ningbo University, 169 Qixinnan Road, Meishan, Beilun District, Ningbo 315211, China; xiong167812@163.com (J.X.); tang97926@163.com (Z.T.); gshyf114204@163.com (Y.Z.); me2445765674@163.com (K.X.); 2 School of Medicine, Ningbo University, 818 Fenghua Road, Jiangbei District, Ningbo 315211, China

**Keywords:** consumption behaviours, COVID-19 pandemic, influence and change, driving determinant, China

## Abstract

This study investigated changes of individuals’ consumption behaviours during the COVID-19 pandemic and explored the driving determinants in consumption expenditure in Zhejiang China. Based on the 454 samples of survey data, which were collected in 2020 and 2021, it showed a reduction trend in consumption expenditure during the pandemic. Compared to the consumptions before the pandemic, money spent on housing, food, and beverage did not change too much. However, expenditures on wearing, recreation, and education reduced. Age, family size, and household income were significant to the expenditure changes. Online shopping became an important alternative way for residents during the pandemic and the trend is expected to continue even after the pandemic. Based on the findings, suggestions are summarized as two points. First, the young and single residents are the main group for recovering the consumption for wearing, recreation, education, and public transport. Meanwhile, to improve the satisfactions in online shopping, regulations should be issued by the government in improving the quality of goods and service.

## 1. Introduction

Since the outbreak of the pandemic caused by COVID-19, there has been a huge influence on the ways of living of residents worldwide. The pandemic has taken away millions of lives. According to the data of World Health Organization, more than 136,291,755 confirmed cases have been monitored and more than 2,941,128 people died during this pandemic by April, 2021. In order to control the spread of the pandemic, measures such as the lockdown of a city or some districts have been taken in different counties. The implementation of these measures has great effects on controlling the spread of pandemic and protecting the health of residents. However, it caused huge economic loss because of movement restrictions of production materials and workers. As a result, the economy grows negatively in many countries. According to the estimation of the International Monetary Fund (IMF), Gross Domestic Product (GDP) of worldwide decreased by 3% in 2020. Major economy countries such as US show a negative growth rate of −5.9% in 2020 [1]. The economy loss caused by the pandemic reaches 158 billion Euro in French in 2020 [2]. Due to the spread of the pandemic and strict control polices for travel activities, the economy loss is expected to continue which would bring great pressures on both residents and governments.

The pandemic of COVID-19 also has a great impact on China’s economy. Almost all fields have been influenced, especially transportation, tourism, catering, etc. Both sales and incomes in these fields decreased. To overcome the challenges, the Chinese government introduced a series of policies to stimulate the domestic consumption. Goals such as expanding consumption scale, raising consumption level, and improving consumption structure, have been set to promote the consumption [3].

To achieve the goals, it is essential to understand the consumers’ behaviours in the pandemic to create effective policies. The outbreak of the pandemic not only greatly impacted on people’s psychology and physical integrity but also changed people’s lifestyle and living habits [4]. With the support of technologies, consumption behaviours have been changed during the pandemic period by many new business modes such as online shopping and non-contact delivery services. For example, online medical market grows from CNY 67.98 billion in 2019 to CNY 94.05 billion in 2020 [5]. Meanwhile, online retail shopping sales increased by 5% in 2020 [6]. Changes of consumption behaviours have great influence on success of policies making aimed to promote consumption in the pandemic. Therefore, investigation of consumption behaviours is essential.

To give suggestions for the policy makers in recovering consumption thus economy, this study aims to investigate the resident consumption behaviours through surveys in China. Analyses based on the surveys are expected to not only reveal the changes of consumption behaviours in the pandemic but also to explore the driving determinants. Findings could lay theoretical understanding of special consumption behaviours in a big pandemic. Moreover, driving determinants identification would provide suggestions for the policy making which aims to recover consumption in practice.

## 2. Literature Review

During the outbreak of the COVID-19 pandemic, consumption behaviours have changed greatly. Research which focuses on consumption behaviours is classified to two types. The first type investigated the consumption of many kinds of goods during the COVID-19 pandemic [7,8,9,10]. For example, Peluso et al. found consumers spend more on sustainable products in an empirical study in Italy [11]. Based on the data from 1548 individuals, Li et al. found impulse consumption increased as the pandemic became worse in China [12]. In Brazil, residents’ alcoholic consumption increased by 44.9% and cigarette consumption increased by 53.6% for 468 residents [13].

The other type of research investigated the consumption of a certain kind of goods during the pandemic of COVID-19 [14,15,16]. Food consumption is a hot topic. Husain and Ashkanani found people have more late-night snacks during the COVID-19 pandemic based on the survey data of 415 adults in Kuwait [7]. However, the consumption of fast-food consumption decreased. Based on an electronic questionnaire, Baaran and Purut found dairy and breakfast products, vegetables, fruits, and nutritional supplements consumption increased during the pandemic. Consumption of bread, meat products, and beverage decreased [17]. In Dutch, Romeo-Arroyo et al. found snacks and ultra-processed food consumption increased in the COVID-19 pandemic based on a national survey of 600 volunteers [18]. In the UK, Coulthard et al. found that the consumption of high energy density snack foods, home prepared foods, and fruits and vegetables increased [19]. However, Faour-Klingbeil et al. found a significant reduction of ready to use food consumption during the pandemic in Lebanon. The percentage of respondents who think they do not order hot and cold ready to use food delivery increase by 19.2% [20]. Compared to food consumption, Lim and To found gambling revenues have decreased in Macao during the pandemic, which could signal a decreased in entertainment and leisure activities [21].

Resource consumption is also an interesting topic during the outbreak of the COVID-19 pandemic. For example, Geraldi et al. found that the electric energy consumption by health centres, administrative buildings, elementary schools, and nursery schools reduced by 11.1%, 38.6%, 50.3%, and 50.4%, respectively [22]. In Joinville, Kalbusch et al. found that the water consumption by the commercial, industrial and public categories decreased. However, water consumption by residential category increased during the pandemic [23].

Findings of above studies showed that the consumption, especially individual consumption has been changed in the pandemic. Most previous studies focused on one or two types of consumption, which fails to detect the change of all consumption behaviours in daily life of residents. It is difficult to lay a strong theory foundation for policy making just based on limited types of consumption. Therefore, it is necessary to investigate multiple consumption behaviours of residents in the pandemic of COVID-19. This study focuses on multiple consumption behaviours of residents from the aspect of consumption expenditure, rather than consumption demand. Data of expenditures are easier to collect but can reflect the amount of consumption indirectly. Moreover, the consumption revenue reflected in expenditure could provide great suggestions for policy makers in aiming to recover the consumption in China.

## 3. Method and Material

### 3.1. Survey

The ‘during and after’ approach was applied, as suggested by Lim [24]. A comparison of consumption behaviours before and during the pandemic can be helpful in delineating the causal effects of the COVID-19 pandemic. We not only investigated the difference in consumption behaviours before and during the pandemic but also assessed the change in consumption behaviours at different stages of the pandemic, such as the epidemic peak and stable control stage. Therefore, two surveys were conducted at two different stages. The first survey was conducted in March 2020, during which the pandemic was at its peak in China. In most Chinese cities, the movement of goods and residents was restricted during this period. The second survey was conducted in March 2021 when the pandemic was controlled. During this period, most cities had implemented mild control polices and had no limitation on travel in the ‘regular epidemic situation’. The questions in both surveys were the same.

Considering the safety and control movement policy, the online surveys were administered on the WeChat platform, which is a popular social app in China. The questionnaire was designed and inputted into ‘Wenjuan Xin’, a professional online survey platform, to generate a QR code. Then, the QR code was shared through WeChat. The respondents scanned the QR code to access the online surveys. They completed the questionnaires online, and the data were automatically stored on the ‘Wenjuan Xin’ platform. In addition, the respondent details such as location and WeChat ID were captured. We predetermined the number of respondents who could complete the surveys. For example, one WeChat ID was limited to only one attempt for completing the survey.

The method of respondent-driven sampling was applied to select subjects in two steps. First, we randomly selected college students living in different cities of Zhejiang province. As online education is the main method for students to learn during the pandemic, all college students have joined many WeChat groups for study purposes. Therefore, selecting students and conducting the survey in these WeChat groups was easy for us. Students who were interested in participating in the survey were included. These students were asked to share the QR Codes with their family members and friends in Zhejiang province for recruiting more subjects. In the two surveys, the number of valid responses obtained was 303 and 151, respectively.

The questionnaire included four parts: basic demographic and socioeconomic information (gender, age, education level, income, occupation, home location, and family size), consumption characteristics before pandemic (consumption type, expenditure, satisfaction, and purchasing channels), consumption characteristics during the pandemic, and consumption expectations after the pandemic.

The questionnaire was dominated by questions. Basically, three types of questions were included in our questionnaire. The first type captured demographic information such as gender, age, education level, income, occupation, home location, family size, and frequency to collect information related to the pandemic, trip mode chosen, consumption type by online shopping, reasons for low satisfaction, measures to promote consumption, means to obtain information related to the pandemic, and means to obtain information related to the pandemic. For this type of question, the percentage of the answer chosen was used as the index to determine the result. The second type of questions was qualitative in nature and captured information such as expenditure on each type of consumption. The data was used directly in the analysis. The third type of questions used the 7-point Likert scale to obtain information regarding the extent of concern or satisfaction of individuals, such as the extent of concern regarding the information related to the COVID-19 pandemic, degree of influence of the COVID-19 pandemic on life, and consumption satisfaction. The scale number was used directly in our paper. Based on the answers, detailed information of the consumption behaviours of the respondents before and during the pandemic was obtained.

### 3.2. Demographic and Socioeconomic Features of Respondents

The demographic and socioeconomic features of the respondents are summarised in Table 1. The distribution of gender in both surveys was balanced. More than a half of the respondents were from cities. More than 70% of the respondents had a bachelor’s or master’s degree. Most respondents were aged from 18 to 40 years. More than 59% of the respondents were single. The annual income of 27.8% of the respondents was less than 24,000 Chinese Yuan (i.e., US $3428 at a currency rate of USD 1 for CNY 7) in 2020; the percentage of the same income group increased to 34.8% in 2021. Most respondents with low incomes were students who received a limited allowance from their family and had limited income from part-time jobs. The majority of the respondents had an income between CNY 48,001 and 100,000 each year.

## 4. Results

### 4.1. Consumption Behaviours before and during the Pandemic

Consumption satisfies the needs of people, which in turn are fulfilled by purchasing and consuming goods and services, such as eating food, travelling, housing, and cleaning clothes [25]. Therefore, the category of consumption could be classified based on the needs of residents. In this study, we classified the consumption into six types according to the basic needs of residents, namely eating and drinking, housing, travel, dressing, recreation and education, and medical healthcare. Clothes, shoes, cleaning products, and laundry service are essential to satisfy the need of dressing, which are called goods for wearing in our study. Therefore, six types of consumption have been indicated as the consumption of food and beverages, housing, traffic trip, goods for wearing, recreation and education, and medical healthcare.

Since it was difficult for the respondents to provide an accurate answer to the quantity of goods, we investigated expenditure rather than the demand for goods. Expenditures for eating and drinking include money spent for buying food, beverage, cigarette, wine, and take-out. Housing expenditure involves the rent or loan payments for houses or apartments. Expenditures for travel include expenses for public and private transport such as cars and e-bike. Money spent on clothes, shoes, hats, make-ups, clothing accessories, cleaning products, and laundry service represents expenditures on goods for wearing. Expenditures for recreation and education include money spent on goods and service for recreation, entertainment, and education. Expenditures for medical healthcare are the money for goods and services related to healthcare, such as medicine, health products, and exercises. Trip mode share was also investigated in detail because travel is a crucial aspect compared with other consumption types during the pandemic.

Consumption behaviours before and during the pandemic in the two surveys are summarised in Table 2. A total of CNY 8309 was spent on the six types of consumption per capita in a month before the pandemic. This consumption expenditure reduced to CNY 6726 during the pandemic in 2020. Housing consumption accounted for 43% of the total expenditures, that is, CNY 3583 and CNY 3119 on average for rent and loan before and during the pandemic, respectively. Expenditures on food and beverages accounted for the second largest share. Although it decreased from CNY 1633 to 1416, the expenditure share of food and beverage increased from 20% before the pandemic to 21% during the pandemic. However, the expenditure on goods for wearing decreased rapidly from CNY 1123 to 757 for each person in one month. Meanwhile, the expenditure share of goods for wearing decreased from 14% to 11%. People also reduced the expenditure for consumption related to recreation and education. Compared with CNY 829 before the pandemic, the expenditure for recreation and education reduced to CNY 531 during the pandemic. People increased the expenditure share of medical healthcare from 5% to 6%. As the total expenditure decreased, money spent on medical healthcare reduced marginally from CNY 398 before the pandemic to CNY 395 during the pandemic.

The mode share of walking, bicycle and e-bike, subway, bus, taxi, and private car were investigated to assess changes in the transport mode choice. In the first survey, private car, walking, and bus were the top three modes used for daily transport. During the pandemic, private transport modes such as private car, walking, and bicycle and e-bike were preferred. Consequently, mode share of public transport such as subway, bus, and taxi, reduced sharply during the pandemic, which can be explained by two reasons. First, choosing private vehicles was believed to be safer as it reduced the chance of coming in contact with other people. Second, public transport was under short supply and strict control during the epidemic peak in 2020, and the operation of the subway and bus had been restricted to control the spread of the virus.

The second survey administered in 2021 revealed that a total of CNY 7976 was spent on the six types of consumption per capita in a month before the pandemic. The total expenditure reduced to CNY 6818 during the pandemic. Housing was the largest part of the expenditure. Respondents spent CNY 2906 and 2951 on an average on housing before and during the pandemic, which was 36% and 43% of the expenditure share, respectively. The expenditures for food and beverages decreased from CNY 1711 to 1189, which constituted 21% and 17% of the total expenditure before and during the pandemic, respectively. Moreover, expenditure on goods for wearing reduced rapidly from CNY 1360 to 853 for each person in 1 month. The expenditure share of goods for wearing decreased from 17% to 13%. Compared with CNY 893 before the pandemic, the expenditure on the consumption of recreation and education reduced to CNY 685 during the pandemic. However, the expenditure on healthcare increased from CNY 302 before the pandemic to CNY 463 during the pandemic, indicating that the respondents had increased their expenditure on medical healthcare during the pandemic.

In the second survey in 2021, bicycle and e-bike, walking, and subway were the top three modes used for daily transport before the pandemic. On the other hand, during the pandemic, the use of subway decreased. Mode share of subway reduced from 16.3% before the pandemic to 13.7% during the pandemic. Instead, residents increased the use of private cars; thus, the mode share of private cars increased from 12.8% to 15.2% during the pandemic.

A reduction in consumption was identified when the consumption behaviours before and during the pandemic were compared based on the data obtained from the two surveys. All types of consumption, especially consumption of goods for wearing and recreation and education, were found to be decreased. However, the opinion differed from the perspective of expenditure share. The expenditure share of medical healthcare increased. It is reasonable for people to spend more on health during a large-scale pandemic. Moreover, the respondents preferred private cars, walking, and bicycle to public transport during pandemic because of safety issues.

### 4.2. Changes in Consumption Behaviours in the Pandemic at Two Time Points

Apart from comparing consumption before and during the pandemic in the two surveys, we analysed changes in consumption behaviours in the pandemic at different time windows. The changes in consumption at the epidemic peak in 2020 and at the stable control stage in 2021 were explored. Considering different social and demographic features of the respondents in the two surveys, the share of expenditure was used to analyse the change in expenditure on consumption.

The changes in consumption during the pandemic in 2020 and 2021 are summarised in Table 3. We noticed an increase in the expenditure share of trips, recreation and education, goods for wearing, and medical healthcare in 2021. Although these expenditures have not recovered to the level similar to that before the pandemic, people are willing to spend on these types of consumption in a stable control situation during the pandemic. Conversely, the expenditure share of housing, food, and beverage has reduced. In the epidemic peak in 2020, the respondents spent more on eating and drinking because of the increased indoor time owing to travel restrictions. Residents also sought cheaper houses to alleviate the pressure of reduced income.

Compared with the trip mode share in 2020, respondents became more confident in using public transport because of operational recovery and strict safe countermeasures such as mandatory mask wearing in 2021. Mode shares of subway, bus, and taxi increased from 6.4%, 1.5%, and 5.7% in 2020 to 13.7%, 13.7%, and 12.4% in 2021, respectively. However, mode shares of walking and private car reduced.

The purchase channel also changed. Online shopping became popular during the pandemic in China because of the movement control policy. The effect on online shopping varied depending on the type of goods. Compared with the data in 2020, shopping activities such as buying clothes, ordering taxis, buying and renting houses online increased in 2021. However, online consumption for fresh food, snacks, and takeaway reduced after reopening of the fresh food market. With operational recovery of public facilities such cinemas, gyms, and schools, a slight decrease in the share of education and recreation and medical healthcare was observed in 2021.

The satisfaction with online consumption was also investigated. The satisfaction with online consumption in 2021 recorded by the respondents was relatively higher than that in 2020. The highest satisfaction was recorded for the online shopping for wearing goods. Increased satisfaction with the online shopping for goods for wearing and online recreation was recorded in 2021. Satisfactions with the online shopping for fresh food and online education also increased; nearly 28.3% of the respondents indicated low satisfaction because of slow delivery. A few respondents indicated items and troubles with return and exchange of goods as important factors for low satisfaction in 2020. On the other hand, customers complained that trouble with return and exchange decreased their satisfaction in 2021. To improve consumer satisfaction, online shops should solve these problems, particularly the trouble in return and exchange of goods and slow delivery. Moreover, attracting more online shops to increase alternative goods and service on the online platform seems a better option.

Regarding the policies that the Chinese government introduced to promote consumption, 38.3% of the respondents in 2020 supported living subsidy to be a good measure to promote consumption. However, the support rate reduced to 33% in 2021. With a stable control of the pandemic, the respondents preferred the pricing policy, which limited the price of certain types of goods. As price fluctuation is common during the pandemic, the government should adopt strict measures to maintain the price of goods, especially of rice, flour, and edible oil. Additionally, consumption coupons were welcomed as an effective way to promote consumption only by 13.2% of the respondents in 2020 and 9.6% of the respondents in 2021.

### 4.3. The Expected Consumption Behaviours in the Future

**Figure 1:**
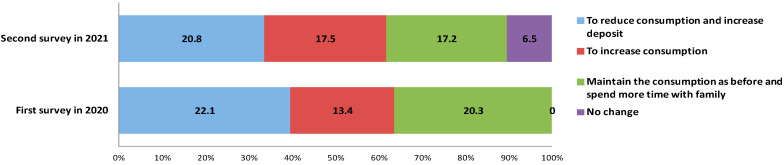
Expected consumption behaviours after the pandemic.

To investigate the effect of the pandemic on consumption in the future after the pandemic, we determined the consumption expectations of the respondents. Figure 1 illustrates the detailed information. Approximately 22.1% and 20.8% of the respondents expressed their willingness to reduce consumption after the pandemic in the first and the second survey, respectively. They believed that saving money is necessary because a high saving balance could ensure safety during an emergency such as the pandemic. Conversely, 13.4% of the respondents in 2020 and 17.5% of the respondents in 2021 stated that they would increase consumption after the pandemic. On the other hand, 20.3% of the respondents in the first survey and 17.2% of the respondents in the second survey responded that they would maintain the consumption as before but spend more time with their family members after the pandemic. Approximately 6.5% of the respondents replied that their consumption behaviour will not change after the pandemic.

Table 4 summarises the expected changes in consumption expenditure after the pandemic based on the two surveys. Most respondents stated that they would maintain a stable expenditure on housing. Interestingly, the number of respondents who are expected to increase the expenditure on all consumptions, except housing after the pandemic was more in the first survey. For example, people believe that health is the most crucial aspect and that they would spend more money on healthcare. Moreover, they stated that they will increase the consumption of recreation and clothes to compensate for the reduced consumption during the pandemic. Consumption expectations in the second survey were different from those in 2020. More people admitted that they would increase consumption on food, beverage, and healthcare but decrease the consumption on travels, wearing goods, recreation, education, and healthcare after the pandemic.

## 5. Discussion

A thorough investigation of the factors causing changes in consumption behaviours is critical for policy-making. Generally, two major factors have been identified, namely features of goods (e.g., choice of goods, availability, price, and quality) and socioeconomic factors of consumers (e.g., gender and income). In this study, we investigated the effect of socioeconomic features of consumers on consumption behaviours.

### 5.1. Socioeconomic Factors and Expenditure Difference before and during the Pandemic

The effect of socioeconomic factors on the change in consumption behaviours was investigated through analysis of variance (ANOVA). Socioeconomic features of consumers included gender, age, education background, occupation, family size, household income, and home location. Changes in consumption behaviours were reflected by the expenditure difference, which is calculated by subtracting the expenditure during the pandemic and the expenditure before the pandemic for each respondent. Six types of expenditure differences were investigated based on the consumption type. Results of ANOVA performed using SPSS software are summarised in Table 5.

The expenditure difference on housing was not significant among the groups having differences in socioeconomic factors. However, the traffic expenditure among respondents who were of different ages and residing at different locations was found to differ significantly. Travel expenditure changed because of the travel control policy, especially for the young people in the city, during the pandemic. Compared with the elderly people living in the countryside who always stay indoors, the young ones in cities had more active trips before the pandemic. Therefore, the pandemic caused a significant effect on the travels of young ones. However, the socioeconomic factors of the respondents were not found to have a significant effect on the expenditure change for food and beverages. Gender and age were the two factors that significantly affected the expenditure on goods for wearing. Compared with men, women tend to buy more goods for wearing such as clothes, shoes, clothing accessories, and housing clean products. The role of women in the family as a house keeper makes them the main consumer of goods for wearing. Moreover, the young individuals are the active consumers of goods for wearing, especially clothes, shoes, and make-up items, because of their greater social interactions. During the pandemic, the face-to-face interactions were limited, which markedly reduced the need for formal dressing. Age caused a significant effect on the expenditure difference of recreation and education. The young individuals were more likely to have recreation and education before the pandemic; the effect of pandemic was more profound on them because of movement restrictions. Educational background, family size, and household income were found be the three factors that significantly affected the expenditure on healthcare. Concerns for health and affordability of healthcare allowed respondents having a good educational background and high income to spend more on health care. Additionally, large families tended to increase the expenditure on healthcare during the pandemic to protect their children.

### 5.2. Socioeconomic Factors and Change of Travel Mode Share before and during the Pandemic

The effect of socioeconomic factors on the travel behaviours was investigated using ANOVA. Socioeconomic features included gender, age, educational background, occupation, family size, household income, and home location. Change in travel behaviours was reflected by the mode share difference, which is calculated by subtracting the mode share during the pandemic and mode share before the pandemic for each respondent. Six types of modes were investigated. Table 6 presents the results of ANOVA performed using SPSS software.

Socioeconomic factors were not found to produce a significant effect on mode share of walking and taxi. However, the mode share on bicycle and e-bike before and during the pandemic among respondents who had different household incomes and home locations was found to differ significantly. A negative relationship was observed between the household income and mode share of bicycle and e-bike; the higher the income, the smaller was the difference. The rich own private cars, and they did not prefer bicycles and e-bikes for their travel before the pandemic. During the pandemic, private cars were their first choice owing to the convenience and safety. People having less income had been using bicycles, e-bikes, bus, and subway as the travel modes before the pandemic; however, during the pandemic, the operation of public transport was restricted, due to which they turned to bicycles and e-bikes only. Therefore, the mode share of bicycles and e-bikes for the low-income respondents, especially those with low incomes in the city, was significantly increased.

Occupation caused a significant effect on the usage of subway during the pandemic. Compared with the mode share before the pandemic, the subway usage among workers in private companies and employees in state-owned companies, government, and public institutions was significantly affected during the pandemic. These occupations have strict work timings, and most employees engaged in these occupations choose the subway to ensure punctuality. During the pandemic, they had to choose other travel modes because of the limited operation of the subway and public safety concerns. However, the respondents comprising college and graduate students, self-employed, jobless, and housewives demonstrated less mode share change for the subway; these respondents were not dependent on the subway because they had a flexible time schedule that allowed them to access more travel choices.

Age and family size were found to have a significant effect on the mode share of the bus. Buses are preferred by the young people and were the main mode before the pandemic. However, the use of buses reduced during the pandemic because of limited service availability and safety concerns. Compared with the young individuals, the pandemic had a less significant effect on the older people who had less bus trips even before the pandemic. The mode share of the bus was also significantly affected by the family size. During the pandemic, bus usage reduced with an increase in the family size. Unmarried people who are also young had been using buses as the main travel mode before the pandemic. For large families with children, buses are not the first choice. Family size was also found be a significant factor for the use of private cars. Small families such as unmarried people who had been using public transport before the pandemic had to substitute the public transport trips by other mode trips during the pandemic. Larger families that have children preferred using private cars both before and after the pandemic, and the pandemic was found to have a little effect of their choice of private car trips.

### 5.3. Concerns Regarding the COVID-19 Pandemic and the Effect on Daily Life

Information of the COVID-19 pandemic has affected the psychology of the residents and eventually of their life and consumption patterns. We aimed to explore the questions such as what information the respondents are concerned about, how to obtain information, what is the frequency of searching information, and to what extent the pandemic has influenced the life of residents. The 7-point Likert scale was used to reflect the concern degree to the information related to COVID-19 pandemic and the degree of influence of the COVID-19 pandemic on life. As shown in Table 7, residents paid considerable attention towards all the information related to the pandemic, with health being the highest priority. In addition, the respondents were sensitive to the recovery policy of normal working and schooling. Travel restrictions and lockdown or reopening the city were also the main concerns. Respondents paid least attention to recreation. Regarding the channels for obtaining information, more than 43% of the respondents obtained information through the internet, while more than 25% of the respondents obtained information through television. The phone was also used to share information during the pandemic. Public broadcasting and mail were not popular for information sharing. The respondents exhibited different frequencies for searching information at two time windows. In the first survey during the epidemic peak, more than 86% of the respondents searched for information at least once a day; however, only 72% of the respondents exhibited this frequency in the second survey. Most respondents believe that the COVID-19 pandemic has a relatively high impact on their everyday life. Travel and social contact have been mostly affected. Intensity and mode of work, psychological safety, exercise, and health keeping have also been affected. Overall, the effect of the pandemic on everyday life was found to be less in the second survey.

**Figure 2:**
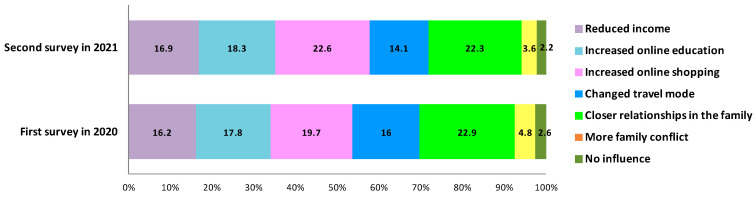
The effect of the pandemic on daily life of residents.

The pandemic has affected many aspects of everyday life and future consumption behaviours of residents in China. Figure 2 illustrates these effects in detail. First, the pandemic had an effect on the income; more than 16% of the respondents indicated decreased income because of unemployment or less opportunities caused by movement restrictions. Second, the pandemic promoted the usage of online shopping; more than 17% and 19% of the respondents reported to have increased their dependency on online education and online shopping, respectively, in the first survey in 2020, whereas in 2021, these rates increased to 18.3% and 22.6%, respectively. These data indicate that an increasing number of people have accepted online shopping and that it has become an alternative shopping mode. Third, 16% of the respondents in the first survey and 14.1% of respondents in the second survey reported that the pandemic has changed their travel mode choice. Fourth, the pandemic has affected the psychology of the people and thus family relationships; during the epidemic peak in 2020, 22.9% of the respondents reported to have close relationships with family members, whereas 3.6% of the respondents reported to have more family conflicts. Compared with the data in 2020, 22.3% of the respondents reported having better family relationships, while 3.6% of them reported having more family conflicts in 2021. Approximately 2.6% and 2.2% of the respondents, respectively, reported that the pandemic had no effect on their life in 2020 and in 2021.

## 6. Conclusions

The pandemic of COVID-19 dramatically impacts the consumers’ consumption behaviours. Analyses on the changes of these behaviours may bring clues for the policy making in promoting the consumption. This paper presented a case study in China to investigate the individuals’ consumption behaviours in the pandemic and driving determinants of consumption expenditure changes. Based on the 454 samples of survey data, which were collected in 2020 and 2021 separately, four major findings were identified. First, health and recovery of work and schooling were most concerned by respondents who indicated the pandemic had most significant influence on travel and social contact, intensity, and mode of work. Second, compared to consumption before the pandemic, there was a decrease of expenditure on consumption during the pandemic. Expenditure share of medical health care increased. Expenditures on consumption of goods for wearing, recreation, and education decreased in the pandemic. Third, compared to high usage of public transport before the pandemic, residents preferred private vehicles, walking, bicycle during the pandemic. Fourth, online shopping became a popular shopping mode in China during the pandemic. Slow delivery, limited choices of items, and hassles in return and exchange of goods are the most important problems that need be solved to improve the satisfaction of online shopping.

Based on the ANOVA results, socioeconomic characteristics of individuals have an influence on the consumption expenditure and travel mode choice. The young and single would like to reduce the expenditure on goods for wearing, recreation and education. To keep a basic living standard in the condition of reduced income in the pandemic, their expenses focused on the consumption of food, housing, and travel. As a result, there was a decreasing consumption market in the retailing, recreation, and education. According to a report by National Statistics Bureau of China, the revenues for the retail shops, restaurants, and hotels reduced by 17.6%, 43.1%, and 50% in 2020, respectively [26]. Meanwhile, those outdoor consumptions were expected to increase as a result of reactance in regaining freedom when lockdown measures are lifted. Policies should be made to guide the satisfaction of this kind of needs accordingly [27].

Based on the findings, suggestions could be put forward. First, policies for improving consumptions could focus on retailing, recreation, and education. The young and singles could be taken as main stream to recover the market of retail, recreation, education, and public transport usage. Second, regulations from government should be issued and be strictly implemented to improve the service of online shopping. As online shopping becomes more and more popular, it is essential to develop policies to ensure benefits of consumers and to improve satisfaction. Last, policies combined living subsidies and reduced price of goods are suggested as an effective way to stimulate the consumption of residents.

Consumption behaviours include not only purchase results, but also purchase process. This paper focuses on qualitative data of expenditure to understand purchasing decisions in beginning. Further research is required to investigate the purchase process of consumption, for example how, where, when, and why each kind of consumption individuals, to complete a full understanding of consumption behaviours and provide more suggestive policies.

## Figures and Tables

**Table 1:** Basic social demographic information of respondents.

Variable	Attribute	Percentage (%) at First Survey	Percentage (%) at Second Survey
Gender	Male	45.9	60.7
	Female	54.1	39.3
Age	18–22 years	22	18.7
	23–29 years	39.3	56.3
	30–40 years	21	10.7
	40–50 years	11.5	11.6
	50–60 years	5.9	2.7
	More than 60 years	0.3	0
Education	Middle school	6.6	21.4
	College and Bachelor	65.6	50
	Master	19.3	25.9
	Doctor	8.5	2.7
Occupation	Workers in private companies	20.3	26.8
	Employee in state-owned companies, government, and public institutions	37.4	21.4
	College and graduate students	35.4	32.1
	Self-employed	5.2	11.6
	jobless or house wives	1.6	8
Family size	Single	59	69.6
	Married without child	3.6	4.5
	Married with one child	25.9	11.6
	Married with two children	11.5	14.3
Home location	City	67.5	59.8
	Countryside	32.5	40.2
Household income	CNY < 24,000/year	27.8	34.8
	CNY 24,000–48,000/year	9.2	8.1
	CNY 48,001–100,000/year	26.6	34.8
	CNY 100,001–240,000/year	23.3	16.1
	CNY > 240,000/year	13.1	7.2

**Table 2:** Consumption expenditure and trip choice before and during the pandemic in two surveys.

**First Survey in 2020**	**Before the Pandemic**		**During the Pandemic**	
Expenditure (Yuan/month)	Housing	3583 (43%)	Housing	3119 (46%)
	Traffic trip	743 (9%)	Traffic trip	508 (8%)
	Food and beverages	1633 (20%)	Food and beverages	1416 (21%)
	Goods for wearing	1123 (14%)	Goods for wearing	757 (11%)
	Recreation and education	829 (10%)	Recreation and education	531 (8%)
	Medical health care	398 (5%)	Medical Health Care	395 (6%)
Trip mode (share %)	Walking	18.5%	Walking	32.4%
	Bicycle, e-bike	15.7%	Bicycle, e-bike	17.1%
	Subway	16.4%	Subway	6.4%
	Bus	18.4%	Bus	1.5%
	Taxi	9.2%	Taxi	5.7%
	Private car	21.8%	Private car	29.9%
**Second Survey in 2021**	**Before the Pandemic**		**During the Pandemic**	
Expenditure (Yuan/month)	Housing	2906 (36%)	Housing	2951 (43%)
	Traffic trip	804 (10%)	Traffic trip	677 (10%)
	Food and beverages	1711 (21%)	Food and beverages	1189 (17%)
	Goods for wearing	1360 (17%)	Goods for wearing	853 (13%)
	Recreation and education	893 (11%)	Recreation and education	685 (10%)
	Medical health care	302 (4%)	Medical Health Care	463 (7%)
Trip mode (share %)	Walking	19.0%	Walking	22.3%
	Bicycle, e-bike	21.1%	Bicycle, e-bike	22.7%
	Subway	16.3%	Subway	13.7%
	Bus	18%	Bus	13.7%
	Taxi	12.8%	Taxi	12.4%
	Private car	12.8%	Private car	15.2%

**Table 3:** Comparison of consumption behaviours during the pandemic in 2020 and in 2021.

Variable	First Survey in 2020		Second Survey in 2021	
Expenditure share (%)	Housing	46%	Housing	43%
	Traffic trip	8%	Traffic trip	10%
	Food and beverages	21%	Food and beverages	17%
	Goods for wearing	11%	Goods for wearing	13%
	Recreation and education	8%	Recreation and education	10%
	Medical Health Care	6%	Medical Health Care	7%
Trip mode (%)	Walking	32.4%	Walking	22.3%
	Bicycle, e-bike,	17.1%	Bicycle, e-bike,	22.7%
	Subway	6.4%	Subway	13.7%
	Bus	1.5%	Bus	13.7%
	Taxi	5.7%	Taxi	12.4%
	Private car	29.9%	Private car	15.2%
Online shopping (%)	Clothes	29.3%	Clothes	29.9%
	Fresh food	14.1%	Fresh food	11.5%
	Snacks and take out	22.4%	Snacks and take out	21.7%
	Taxi order	10.0%	Taxi order	12.1%
	Education and recreation	15.3%	Education and recreation	15.0%
	Medicine	5.9%	Medicine	5.7%
	House sell and rent	3.0%	House sell and rent	4.1%
Consumption satisfaction (highest—7, lowest—1)	Online shopping of fresh food	4.22	Online shopping of fresh food	4.36
	Online shopping of	4.51	Online shopping of	4.71
	Goods for wearing		Goods for wearing	
	Online education	4.35	Online education	4.36
	Online recreation	4.43	Online recreation	4.66
Reasons for low satisfaction (%)	Few choices of items	23.4%	Few choices of items	17.6%
	Slow delivery	28.3%	Slow delivery	21.8%
	Low quality of goods	16.2%	Low quality of goods	18.8%
	Trouble with return and exchange	21.2%	Trouble with return and exchange	20.9%
	No comparison	10.9%	No comparison	20.9%
Measures to promote consumption (%)	Consumption coupon	13.2%	Consumption coupon	9.6%
	Reduced price of goods	25.1%	Reduced price of goods	29.6%
	Living subsidies	38.3%	Living subsidies	33.0%
	Controlled price of certain goods	23.4%	Controlled price of certain goods	27.8%

**Table 4:** Expected change of consumption expenditure after the pandemic.

	First Survey in 2020			Second Survey in 2021		
Consumption	Increase	Unchanged	Decrease	Increase	Unchanged	Decrease
Housing	10.2	76.9	12.9	13.3	67.0	20.0
Travel	32.7	47.2	20.1	22.6	47.0	30.4
Food and beverage	31.0	45.2	23.8	25.2	57.4	17.4
Goods for wearing	31.7	45.9	22.4	24.3	46.1	29.6
Recreation and education	32.3	41.6	26.1	23.5	46.1	30.4
Health care	35.6	47.2	17.2	31.3	45.2	23.5

**Table 5:** Result of ANOVA for socioeconomic factors and change of expenditure before and during the pandemic.

Expenditure Difference	Housing		Traffic		Food and Beverage		Good for Wearing		Recreation and Education		Health Care	
	F	Sig	F	Sig	F	Sig	F	Sig	F	Sig	F	Sig
Gender	1.34	0.25	0.33	0.56	0.14	0.71	3.73	0.05 *	2.20	0.14	2.94	0.08
Age	0.85	0.52	2.33	0.04 *	0.28	0.92	2.30	0.04 *	2.80	0.01 **	1.06	0.38
Education background	0.10	0.96	1.49	0.24	0.06	0.98	0.55	0.65	1.42	0.23	2.89	0.03 *
Occupation	0.4	0.81	0.23	0.92	1.15	0.33	0.32	0.86	0.51	0.68	0.62	0.65
Family size	0.33	0.80	2.15	0.09	0.50	0.68	1.58	0.20	2.29	0.07	3.95	0.01 **
Household income	0.13	1.00	1.09	0.32	0.60	0.98	0.51	1.00	0.74	0.89	7.19	0.00 **
Home location	0.44	0.51	6.81	0.01 **	0.22	0.64	0.67	0.41	0.13	0.72	0.25	0.62

Note: * significant level at 0.05, ** significant level at 0.01.

**Table 6:** Result of ANOVA for socioeconomic factors and mode share difference before and during the pandemic.

Mode Share Difference	Walking		Bicycle and e-Bike		Subway		Bus		Taxi		Private Car	
	F	Sig	F	Sig	F	Sig	F	Sig	F	Sig	F	Sig
Gender	0.34	0.56	0.33	0.57	0.02	0.89	1.08	0.3	0.11	0.74	0.95	0.33
Age	0.95	0.48	0.57	0.72	1.73	0.12	2.55	0.03 *	0.45	0.81	1.36	0.24
Education background	0.66	0.58	0.43	0.73	1.13	0.34	2.13	0.09	0.74	0.53	2.41	0.06
Occupation	0.71	0.58	0.25	0.91	2.77	0.03 *	2.03	0.09	0.72	0.58	2.11	0.08
Family size	1.02	0.38	0.63	0.60	2.17	0.09	4.82	0.00 **	0.40	0.76	3.68	0.01 *
Household income	1.01	0.46	1.62	0.01 *	1.33	0.08	0.56	0.99	1.23	0.16	1.21	0.18
Home location	0.03	0.85	2.46	0.02 *	0.40	0.52	0.56	0.45	0.01	0.98	0.76	0.38

Note: * significant level at 0.05, ** significant level at 0.01.

**Table 7:** Concerns regarding the COVID-19 pandemic.

Variable	At First Survey		At Second Survey	
Concern degree to the information related to COVID-19 pandemic (highest level is 7 and lowest level is 1)	Lockdown or open city	5.11	Lockdown or open city	5.09
	Travel control	5.16	Travel control	5.17
	Household goods shopping	4.94	Household goods shopping	4.61
	Recovery of work and schooling	5.29	Recovery of work and schooling	5.28
	Recreation	4.43	Recreation	4.38
	Health	5.46	Health	5.58
Ways to obtain information related to the pandemic (%)	Internet	45.1	Internet	43.4
	Broadcasting	8.1	Broadcasting	12
	Television	30.5	Television	25.3
	Phone	15.2	Phone	16.9
	Mail	1.1	Mail	2.4
Frequency to collect information related to the pandemic (%)	Once a couple of hours	33.7	Once a couple of hours	36
	Once a day	52.8	Once a day	36
	Once two or three days	12.2	Once two or three days	22.5
	Once a week	1.3	Once a week	3.7
	Once a month	0	Once a month	1.8
Degree of influence of the COVID-19 pandemic on life (highest degree is 7 and lowest degree is 1)	Intensity and mode of work	4.85	Intensity and mode of work	4.57
	Income	4.11	Income	4.15
	Consumption amount	4.45	Consumption amount	4.17
	Shopping mode	4.32	Shopping mode	4.31
	Travel and social contact	4.93	Travel and social contact	4.89
	Exercise and health keeping	4.65	Exercise and health keeping	4.46
	Psychological safety	4.65	Psychological safety	4.72
